# Ovarian high-grade serous carcinoma with elevated β-human chorionic gonadotropin: A case report

**DOI:** 10.1097/MD.0000000000028144

**Published:** 2021-12-23

**Authors:** Yi Zhong, Xing Chen, Yan Huang, Yi Jiang, Ting Chen, Xianglin Nie, Wenjun Cheng

**Affiliations:** a Department of Gynecology, the First Affiliated Hospital with Nanjing Medical University, Nanjing, China; b Department of Women Health, Zhangqiu Women and Children Health Hospital, Zhangqiu District, Ji’nan, China; c Department of Radiology, the First Affiliated Hospital with Nanjing Medical University, Nanjing, China.

**Keywords:** β-human chorionic gonadotropin, chemotherapy, metastasis, ovarian serous carcinoma, prognosis

## Abstract

**Rationale:**

Human chorionic gonadotropin (hCG) is a glycoprotein hormone secreted by the syncytiotrophoblasts of the placenta. However, hCG (particularly β-hCG) is also expressed in many normal nontrophoblastic tissues. Here, we report the case of a 50-year-old woman diagnosed with ovarian high-grade serous carcinoma with elevated β-hCG, which was insensitive to chemotherapeutic drugs and had a poor prognosis.

**Patient concerns:**

A 50-year-old woman with abdominal distention was admitted to our hospital. Pelvic computed tomography and magnetic resonance imaging were highly suggestive of multiple metastases of ovarian cancer. Surprisingly, an elevation in β-hCG levels was also measured.

**Diagnosis and interventions:**

The patient underwent laparoscopic examination and was diagnosed with high-grade serous ovarian carcinoma. After 2 prior chemotherapies with paclitaxel and carboplatin, the patient underwent cytoreductive surgery and continued receiving chemotherapy. However, recurrent lesions were observed during the period of chemotherapy, and the level of β-hCG increased. Alternative chemotherapy with liposomal doxorubicin was administered, but it also had a poor therapeutic effect.

**Outcomes:**

The progression was rapid with a continuous increase in β-hCG levels, and the patient died 9 months after surgery.

**Lessons:**

Gynecologists should be aware of women with ovarian carcinoma with an elevated β-hCG level, which suggests a poor prognosis.

## 1 Introduction

Ovarian cancer is the tenth most commonly diagnosed cancer and the tenth most common cause of cancer-related deaths among women in China.^[1]^ It has been estimated that 52,100 new ovarian cancer cases were diagnosed and 22,500 ovarian cancer deaths in 2015 in China.^[1]^ The prognosis of ovarian cancer is the worst among all gynecological cancers. The main reason for this is that approximately 75% of patients are diagnosed at a late stage because of the asymptomatic characteristics at an early stage.^[2]^ Ovarian cancer have a variety of histological subtypes that have different characteristics. Epithelial cancers comprise nearly 90% of all ovarian cancers. Among the above types, high-grade serous carcinoma is the most common type of epithelial ovarian cancer.^[3]^

Human chorionic gonadotropin (hCG), which consists of an α and β subunit, is a glycoprotein hormone secreted by the syncytiotrophoblast of the placenta. The α subunit is structurally similar to luteinizing hormone (LH), while the β subunit is specific for hCG. Similar to LH, hCG binds to LH/hCG receptors (LHCGRs) to regulate reproductive biological processes. However, the presence of LH/hCGRs has also been confirmed in various nongonadal tissues, such as the cervix, uterus, oviduct, breast, adrenal gland, and brain.^[4]^ Furthermore, hCG (particularly β-hCG) has also been expressed in many normal nontrophoblastic tissues, including pituitary glands, testes, prostate, skeletal muscles, and thymus.^[5]^ Recently, it has been demonstrated that elevated β-hCG is ectopically expressed in a large number of malignant tumor tissues, including ovarian, cervical, endometrial, vaginal, breast, bladder, prostate, gastrointestinal, and lung.^[4,5]^ Here we report the case of a 50-year-old woman diagnosed with ovarian high-grade serous carcinoma with elevated β-hCG, which was insensitive to chemotherapeutic drugs and had a poor prognosis.

## 2 Case presentation

**Figure 1:**
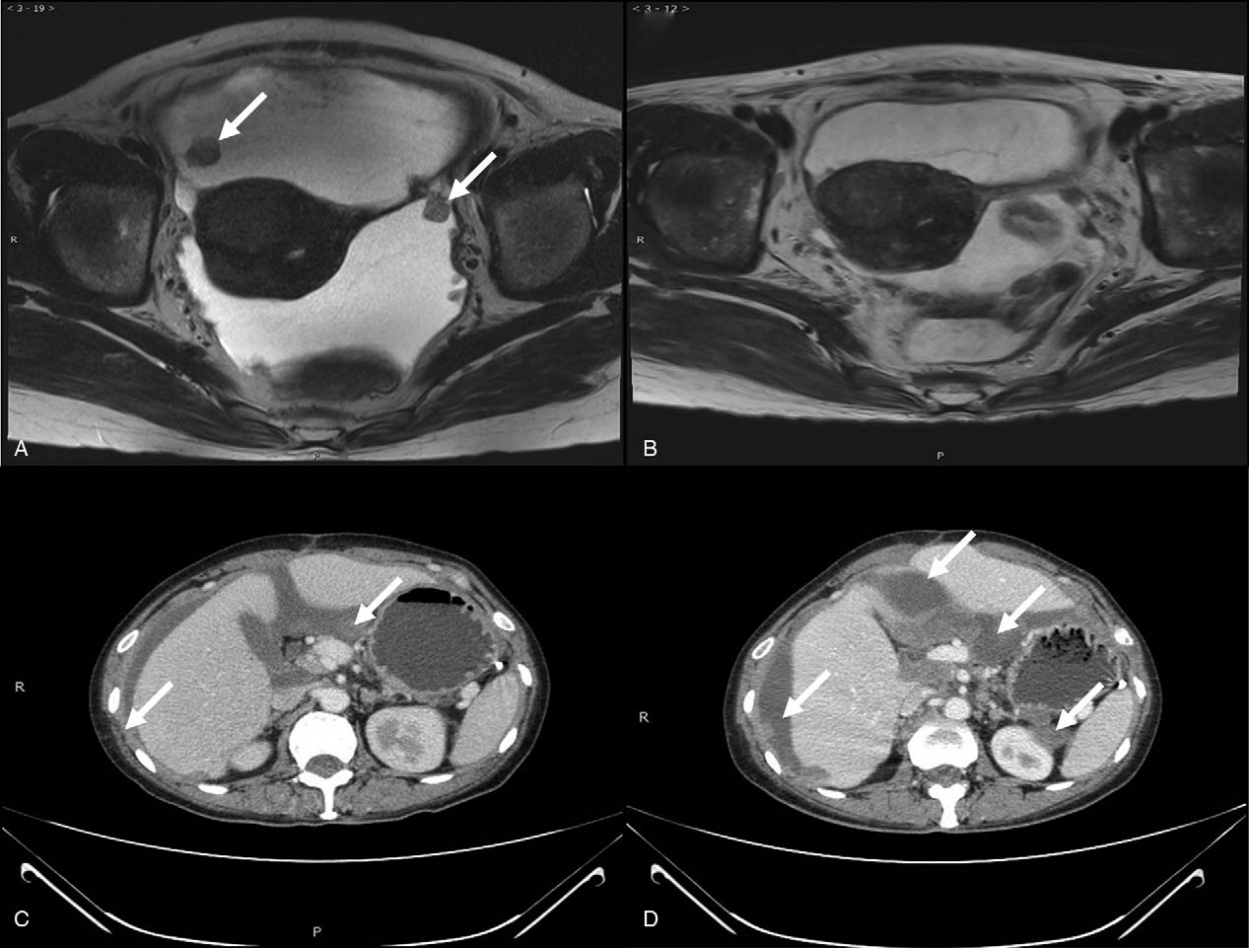
Results of pelvic computerized tomography (CT) and magnetic resonance imaging (MRI) of the patient. (A) Pelvic MRI before treatment which showed large amount of ascites and lesions (white arrows). (B) Pelvic MRI after 2 courses of chemotherapies (paclitaxel plus carboplatin) which demonstrated largely reduced ascites and lesions. (C) Abdominal CT and observed recurrent masses of soft-tissue density in pelvic and liver (white arrows) after the fifth chemotherapy (paclitaxel plus carboplatin). (D) Re-examination of abdominal CT found more and larger lesions after 8 courses of chemotherapies (5 courses of paclitaxel plus carboplatin and 3 courses of liposomal doxorubicin (white arrows).

**Figure 2:**
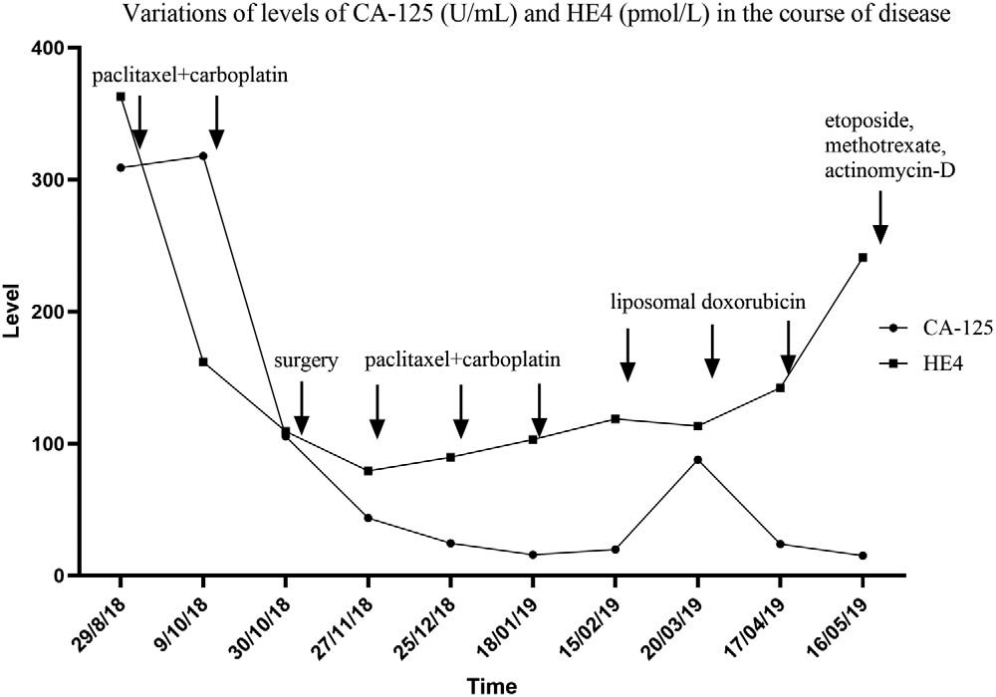
The variation of levels of cancer antigen 125 (CA-125) and human epididymis protein 4 (HE4) in the course of disease (the reference value: CA-125 < 35 U/mL, HE4 < 140 pmol/L).

**Figure 3:**
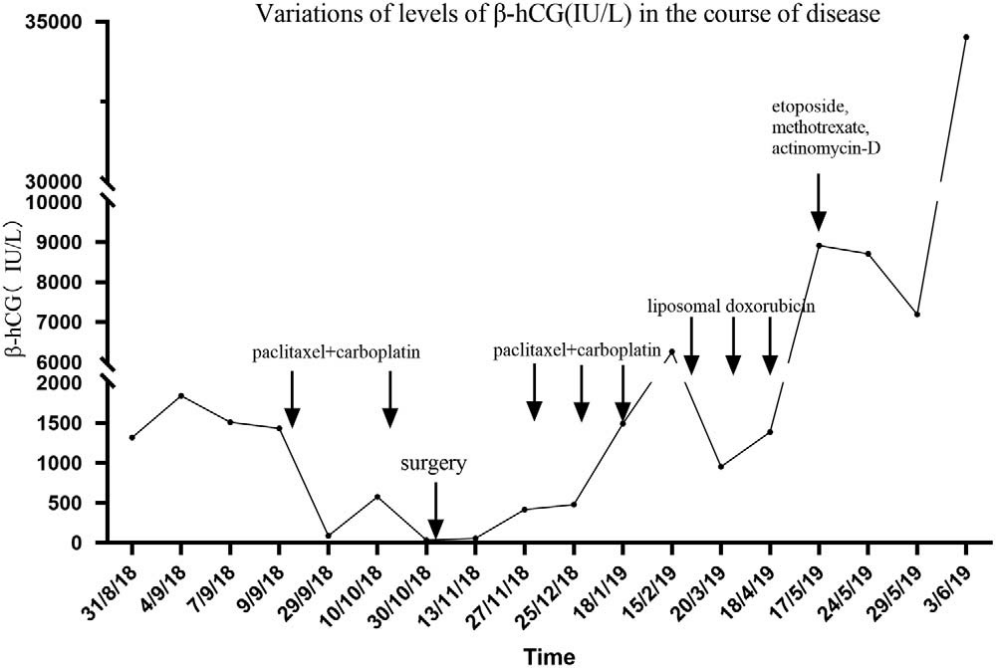
The variation of levels of human chorionic gonadotropin β subunit (β-hCG) in the course of disease.

A 50-year-old woman was referred to our hospital for suspicion of multiple metastases of ovarian cancer on August 29, 2018. She was admitted to a local hospital because of abdominal distention. Ultrasonography revealed a large amount of ascites. Further abdominal and pelvic computed tomography (CT) was performed, and multiple metastases of the peritoneum and omentum were suspected. Laboratory examination revealed elevated levels of cancer antigen 125 (CA-125) and human epididymis protein 4 (HE4) (309.1 U/mL and 363 pmol/L, respectively). Furthermore, a surprising elevation of β-hCG was also found (1315.6 IU/L) on August 31, 2018. Cytology of the ascites exfoliate cells revealed poorly differentiated carcinoma. For further evaluation, she underwent pelvic magnetic resonance imaging and detected massive effusion and enlargement of multiple pelvic and abdominal lymph nodes, as well as abnormal signal intensities in the left adnexa with restricted diffusion on August 31, 2018 (Fig. 1A). On September 6, the patient received laparoscopic examination was performed. Massive ascites and scattered implantation lesions on the uterus, ovaries, fallopian tubes, bladder, intestine, peritoneum, omentum, and diaphragm were observed, which made it impossible to perform satisfactory cytoreductive surgery. Bilateral salpingectomy, ovary, multiple peritoneal biopsies, and curettage were performed. Histopathological examination of all specimens suggested a poorly differentiated carcinoma. Immunohistochemical staining revealed cytokeratin-pan (++), vimentin (-), estrogen receptor (-), progesterone receptor (-), P16 (++), CK7 (++), Pax-8 (+), P53 (++), WT-1 (+), Ki67 (90%+), CK20 (-), and vimentin (-), which confirmed ovarian high-grade serous carcinoma. After laparoscopic examination, the patient received neoadjuvant chemotherapy (paclitaxel 270 mg and carboplatin 450 mg) on September 11 and October 11, 2018. The levels of CA-125 after 2 prior chemotherapies were 318 and 105.5 U/mL while HE4 were 167.9 and 109.3 pmol/L. The serum values of β-hCG after chemotherapies were reduced to 574.6 and 33.9 IU/L, respectively. Repeated pelvic magnetic resonance imaging revealed a significant reduction in ascites and lesions of the abdomen and pelvis (Fig. 1B). Total abdominal hysterectomy, bilateral oophorectomy, paraaortic lymph node dissection, and resection of the tumor lesions were performed on October 31, 2018. The final diagnosis was ovarian high-grade serous carcinoma with paraaortic lymph node metastasis (stage IIIC). The value of β-hCG was 55.2 IU/L after operation on November 13, 2018. The levels of CA-125, HE4, and β-hCG before the third chemotherapy were 43.6 U/mL, 79.4 pmol/L, and 415.4 IU/L. Three courses of chemotherapy (paclitaxel 270 mg and carboplatin 450 mg) were administered on November 29, 2018, December 27, 2018, and January 19, 2019. The levels of CA-125 and HE4 after the 3 chemotherapies were within the normal range, while the levels of β-hCG were 477.7, 1490.4, and 6258.6 IU/L, respectively. Abdominal CT was performed after the fifth chemotherapy and recurrent masses of soft-tissue density in the pelvis and liver (Fig. 1C). The gene test was used to detect mutations of uncertain significance in *FANCA*, *BRCA1*, and *BRIP1*. Considering that the chemotherapy had poor efficiency, liposomal doxorubicin was administered as an alternative chemotherapy 3 times on February 22, March 22, and April 19, 2019. The values of CA-125 after the 3 alternative chemotherapies were 87.9, 23.8, and 15.1 U/mL while those of HE4 were 113.3, 142.4, and 241.0 pmol/L (Fig. 2). The levels of β-hCG after administration of liposomal doxorubicin were 951.4, 1384.3, and 8911.0 IU/L, respectively. Repeated abdominal CT revealed multiple recurrent lesions after 3 courses of liposomal doxorubicin (Fig. 1D). For poor therapeutic effect of liposomal doxorubicin, chemotherapy of etoposide (150 mg d1 + 150 mg d2), methotrexate (150 mg + 300 mg d1), and actinomycin-D (0.5 mg d1 + 0.5 mg d2) (EMA) were administrated on May 17, 2019. Unfortunately, the patient had a fever on May 19, 2019, with negative blood culture results for bacteria and fungi. The temperature ranged from 36.2°C to 38.3°C in 2 weeks, with no efficiency of treatment with cefoperazone and moxifloxacin. The patient was finally diagnosed with a neoplastic fever. The levels of β-hCG were 8709.0 IU/L on May 24, 7191.4 IU/L on May 29 and 34516.4 IU/L on June 3, 2019 (Fig. 3). After sufficient communication with the patient and relatives, they suggested returning to the local hospital for palliative care. The patient finally died 1 month later due to multiple organ failure.

## 3 Discussion

HCG can be measured at a low concentration in the serum of most people, and the value of hCG varies according to the level of gonadotropin-releasing hormone secreted from the pituitary. HCG has been previously found to be ectopically secreted in some nontrophoblastic tumors, including ovarian cancer. HCG has been considered a prognostic biomarker associated with stage and grade in ovarian cancer.^[6]^ Elevated level of serum β-hCG were highly correlated with higher aggressiveness and resistance to various therapies. A study that consisted of 40 ovarian cancer tissues concluded that 68% of patients with ovarian cancer had elevated hCG serum concentrations and were positive for hCG expression in ovarian cancer tissues.^[6]^ HCG tissue expression is highly related to tumor grade, but not histological subtype. Furthermore, mucinous ovarian carcinomas demonstrated remarkably elevated hCG expression at stage III compared to stage I.^[6]^ In this case, the patient was diagnosed with high-grade serous ovarian carcinoma, with a significant increase in the level of β-hCG. It was not sensitive to commonly used chemotherapy drugs and progressed rapidly with a continued increase in β-hCG levels, suggesting that β-hCG was associated with the progression of ovarian cancer. In addition, hCG expression was positively correlated with LHCGR expression in ovarian cancer tissues because hCG could also bind to the LH receptor. In addition to hCG, LHCGR expression was also an independent prognostic factor for the occurrence, progression, and overall survival of patients with ovarian cancer.^[5]^

The mechanisms of hCG in cancer development and progression are not fully understood. Vasculogenic mimicry (VM) is defined as the transformation of aggressive tumor cells to an endothelial cell-like phenotype, thus forming tumor cell-lined vasculature independently, which is independent of host vessels. The presence of VM is highly associated with metastasis and poor prognosis.^[7]^ As a proangiogenic factor, hCG can induce neovascularization in the placenta during pregnancy. In ovarian cancer, it has been proven that hCG improved the formation of VM in the ovarian cancer cell line OVCAR-3 in vivo and in vitro, as well as largely increased the expression of vascular endothelin growth factor, CD31, and factor VIII by activating the LH receptor, thus improving tumor growth.^[8]^ Moreover, hCG also induced the expression of hypoxia-inducible factor-1α, which synergistically regulated tumor VM.^[8]^

In addition, hCG can also inhibit the apoptosis of cancer cells. It has been reported that overexpression of β-hCG (CGB5 gene) in the ovarian cancer cell lines OVCAR-3 and SKOV-3 showed a remarkable decrease in the expression of *BCL2*, but increased expression of *BAX* and BIRC5. Furthermore, a low BCL2/BAX ratio was revealed, thus inhibiting apoptosis.^[9]^ Another in vitro study demonstrated that silencing of CGB genes induces apoptosis in cervical cancer cells.^[10]^ The anti-apoptosis effect may be exerted by transforming growth factor β1 in cancer cells.^[11]^ Furthermore, overexpression of hCG promoted the proliferation of ovarian epithelial T29 and T80 cell lines in vitro, as well as increased G2 cell populations and elevated expression of cyclin E, cyclin D1, and cyclin-dependent kinases 2, 4, and 6, thus regulating the progression of the cell cycle.^[12]^

Epithelial mesenchymal transition (EMT) is defined as polarized epithelial cells that obtain mesenchymal properties that allow them to migrate and invade, which plays an important role in tumor metastasis. During EMT, epithelial cells remodel their cytoskeleton, lose cell–cell junctions, and decrease the expression of epithelial markers such as E-cadherin and zonula occludens-1, leading to increased expression of mesenchymal markers such as N-cadherin, vimentin, and Snail. Overexpression of β-hCG promoted cell migration and invasion in ES-2 and SKOV3 ovarian cancer cell lines by transwell assays and wound healing in vitro and in a nude mouse model in vivo, while silencing β-hCG resulted in the opposite effect.^[13]^ The effect of β-hCG was mediated by the activation of extracellular signal-regulated kinase 1/2 and matrix metalloprotease-2.^[11]^ Overexpression of β-hCG also increased the expression of mesenchymal cell markers and decreased the expression of epithelial cell markers, thus improving EMT and metastasis of ovarian cancer.

Despite huge progress have been made in the fight against ovarian cancer, great challenges remain for advanced, recurrent, and metastatic ovarian cancer. The gene test of this patient showed mutations of uncertain significance in *FANCA*, *BRCA1*, and *BRIP1*, which have been associated with hereditary ovarian cancers. However, the clinical significance of mutations in these genes remains unknown and requires further exploration. Since hCG binds to LHCGRs to regulate biological processes, receptor-mediated therapy might be an alternative for cancer treatment. It has been reported that the effect of hCG-doxorubicin on ovarian cancer cells could increase the activity of doxorubicin and may have better therapeutic effects.^[14]^ However, more effective therapies for cancer with elevated β-hCG levels are imperative.

## 4 Conclusion

In conclusion, elevated β-hCG levels have been observed in various cancer types in the clinic. HCG has been regarded as a prognostic biomarker that is associated with higher aggressive behaviors. However, the mechanism by which β-hCG affects cancer progression needs to be further explored. Therefore, there is an urgent need to develop more effective anticancer therapies to delay progression and improve prognosis.

## Author contributions

YZ and YH wrote the manuscript. YJ, TC, and XN contributed to the data collection and analysis. XC and WC contributed to the data interpretation and critical revision. All authors have read and approved the manuscript.

**Conceptualization:** Yi Zhong, Xing Chen.

**Data curation:** Yi Jiang, Ting Chen, Xianglin Nie.

**Formal analysis:** Yi Jiang, Ting Chen, Xianglin Nie.

**Supervision:** Xing Chen, Wenjun Cheng.

**Writing – original draft:** Yi Zhong, Yan Huang.

**Writing – review & editing:** Xing Chen, Wenjun Cheng.

## References

[1] Chen W Zheng R Baade PD. Cancer statistics in China, 2015. CA Cancer J Clin 2016;66:115–32. 26808342 10.3322/caac.21338

[2] Lheureux S Gourley C Vergote I. Epithelial ovarian cancer. Lancet 2019;393:1240–53. 30910306 10.1016/S0140-6736(18)32552-2

[3] Matulonis UA Sood AK Fallowfield L. Ovarian cancer. Nat Rev Dis Primers 2017;2:16061. 10.1038/nrdp.2016.61 PMC7290868 27558151

[4] Jankowska A Andrusiewicz M Grabowski J. Coexpression of human chorionic gonadotropin beta subunit and its receptor in nontrophoblastic gynecological cancer. Int J Gynecol Cancer 2008;18:1102–7. 18217981 10.1111/j.1525-1438.2007.01151.x

[5] Zhong Y Wang Y Huang J. Association of hCG and LHCGR expression patterns with clinicopathological parameters in ovarian cancer. Pathol Res Pract 2019;215:748–54. 30712886 10.1016/j.prp.2019.01.001

[6] Lenhard M Tsvilina A Schumacher L. Human chorionic gonadotropin and its relation to grade, stage and patient survival in ovarian cancer. BMC Cancer 2012;12:02. 10.1186/1471-2407-12-2 PMC3311592 22214378

[7] Su M Fan C Gao S. An HCG-rich microenvironment contributes to ovarian cancer cell differentiation into endothelioid cells in a three-dimensional culture system. Oncol Rep 2015;34:2395–402. 26479853 10.3892/or.2015.4215

[8] Gao S Fan C Huang H. Effects of HCG on human epithelial ovarian cancer vasculogenic mimicry formation in vivo. Oncol Lett 2016;12:459–66. 27347165 10.3892/ol.2016.4630 PMC4907296

[9] Szczerba A Śliwa A Kubiczak M. Human chorionic gonadotropin β subunit affects the expression of apoptosis-regulating factors in ovarian cancer. Oncol Rep 2016;35:538–45. 26530886 10.3892/or.2015.4386

[10] Jankowska A Gunderson SI Andrusiewicz M. Reduction of human chorionic gonadotropin beta subunit expression by modified U1 snRNA caused apoptosis in cervical cancer cells. Mol Cancer 2008;7:26–31. 18339208 10.1186/1476-4598-7-26 PMC2335103

[11] Schüler-Toprak S Treeck O Ortmann O. Human chorionic gonadotropin and breast cancer. Int J Mol Sci 2017;18:1587. 28754015 10.3390/ijms18071587 PMC5536074

[12] Guo X Liu G Schauer IG. Overexpression of the β subunit of human chorionic gonadotropin promotes the transformation of human ovarian epithelial cells and ovarian tumorigenesis. Am J Pathol 2011;179:1385–93. 21763678 10.1016/j.ajpath.2011.05.018 PMC3157261

[13] Liu N Peng SM Zhan GX. Human chorionic gonadotropin β regulates epithelial-mesenchymal transition and metastasis in human ovarian cancer. Oncol Rep 2017;38:1464–72. 28713970 10.3892/or.2017.5818 PMC5549031

[14] Gebauer G Mueller N Fehm T. Expression and regulation of luteinizing hormone/human chorionic gonadotropin receptors in ovarian cancer and its correlation to human chorionic gonadotropin-doxorubicin sensitivity. Am J Obstet Gynecol 2004;190:1621–8. 15284756 10.1016/j.ajog.2004.03.045

